# Towards additive manufacturing oriented geometric modeling using implicit functions

**DOI:** 10.1186/s42492-018-0009-y

**Published:** 2018-09-05

**Authors:** Qingde Li, Qingqi Hong, Quan Qi, Xinhui Ma, Xie Han, Jie Tian

**Affiliations:** 1 School of Engineering and Computer Science, University of Hull, Hull, HU6 7RX UK; 2 Software School, Xiamen University, Xiamen, China; 3 School of Computer Science and Control Engineering, North University of China, Taiyuan, China; 4 Intelligent Bioinformatics Systems Division, Institute of Automation, The Chinese Academy of Sciences, Beijing, China

**Keywords:** Additive manufacturing, 3D printing-friendly CAD, Implicit function, Isosurface, Level-set, Function-based shape modeling, Implicit modeling

## Abstract

Surface-based geometric modeling has many advantages in terms of visualization and traditional subtractive manufacturing using computer-numerical-control cutting-machine tools. However, it is not an ideal solution for additive manufacturing because to digitally print a surface-represented geometric object using a certain additive manufacturing technology, the object has to be converted into a solid representation. However, converting a known surface-based geometric representation into a printable representation is essentially a redesign process, and this is especially the case, when its interior material structure needs to be considered. To specify a 3D geometric object that is ready to be digitally manufactured, its representation has to be in a certain volumetric form. In this research, we show how some of the difficulties experienced in additive manufacturing can be easily solved by using implicitly represented geometric objects. Like surface-based geometric representation is subtractive manufacturing-friendly, implicitly described geometric objects are additive manufacturing-friendly: implicit shapes are 3D printing ready. The implicit geometric representation allows to combine a geometric shape, material colors, an interior material structure, and other required attributes in one single description as a set of implicit functions, and no conversion is needed. In addition, as implicit objects are typically specified procedurally, very little data is used in their specifications, which makes them particularly useful for design and visualization with modern cloud-based mobile devices, which usually do not have very big storage spaces. Finally, implicit modeling is a design procedure that is parallel computing-friendly, as the design of a complex geometric object can be divided into a set of simple shape-designing tasks, owing to the availability of shape-preserving implicit blending operations.

## Background

As envisioned in [1], the next industrial revolution will be about the digitalization of the entire manufacturing process, right from the initial conceptual design, to the manufacturing of the required product in the final stage of the process. Underpinned by artificial intelligence, cyber-physical systems, the internet of things, and cloud computing, this fast approaching revolution raises various challenges to engineers and scientists. As the geometric design is the first step in the process of additive manufacturing (AM), the development of an AM-friendly geometric modeling technique is one of the most important tasks. The geometric objects created by conventional computer-aided design (CAD) techniques are mostly represented by surfaces, which is an ideal solution for the visualization and traditional subtractive manufacturing using computer numerical control (CNC) cutting machine tools, where an object is digitally manufactured by means of drilling, cutting, and slicing. When the main operations of making an object involve drilling, cutting, or slicing, the surface-based representation is sufficient enough, as no interior geometric structure or material properties need to be known. However, the surface-based shape representation is far from being sufficient for AM. This is because the surface representation describes a geometric shape as an infinitely thin boundary object, which does not provide any information required in AM regarding the interior structure of the object, which is to be additively manufactured. Though surface-based representation is suitable for visualization and subtractive manufacturing, there is a large difference between a surface model and the product made from the surface representation. Thus, converting a known surface-based geometric representation into a printable representation is essentially a redesign process.

The design of a geometric model for visualization or for subtractive manufacturing and the design for the AM have completely different requirements: the former activity is mainly concerned mainly with the specification of an object’s external surface details, while the latter one must precisely specify both the external surface details and the internal structure and material details. This is because, when a geometric model is used as an opaque object for visualization or for digital manufacturing based on CNC cutting machine tools, there is no need to know its internal structure, and only surface details are required. However, with AM technologies, an object is progressively built up layer-by-layer, with each layer being a thin-solid slice of the object. To print a layer, for each point on the plane corresponding to the layer, the machine must know whether the given position belongs to the object and what material should be used for printing the point. Obviously, the surface-based geometric representation does not meet this requirement of AM. An ideal 3D printing ready representation for a geometric object should be expressed in a kind of a solid form, which, when printed slice-by-slice, can directly provide clear instructions to the printing machine about where to print. In many ways, solid modeling offers a much better solution when compared with the surface-only representation, as it can directly provide the information about the areas to be printed for each object slice.

Solid modeling can be implemented either explicitly as a collection of voxels and tetrahedra, or as parametric solids, or implicitly as a field function defined in $\mathcal {R}^{3}$. However, representing a solid object as a collection of 3D voxel points or a set of tetrahedra can be expensive in terms of the required storage space. More importantly, they are not an exact representation. Irrespective of the number of voxels or tetrahedra are used, they only provide an approximate solution. Compared with discretely represented solids, parametric solids can provide an exact representation to a solid geometry; however, it is generally difficult to design complex material structures, especially when multiple material structures need to be designed. A natural way to model a ready-to-print geometric object is to represent a geometric object as a 3D function *F*(*x*,*y*,*z*), which can directly inform the printing machine whether a position **P**(*x*,*y*,*z*) should be printed. Some recent research has shown that implicit functions are particularly suitable for modeling microporous structures [2–6]. However, despite its great advantages in modeling geometric objects for the AM, implicit modeling is only used in an ad hoc manner as a supplemental technique. Today, surface-based modeling is still being used as a predominant technique in geometric design, even in the area of AM. In this paper, we intend to show that implicit function-based geometric modeling is in its nature AM-friendly and has an innate advantage over the explicit methods, when the purpose of modeling is to create a geometry for AM rather than for visualization or for subtractive manufacturing using a CNC machine tool. They can be used in general to model any geometric object, much beyond their use in porous structure modeling.

The goal of this study is to show that implicit modeling can play an important role in AM and to promote research on the development of AM-oriented CAD techniques. First, we address the pressing need for the development of AM-oriented CAD techniques, which is followed by a brief introduction to implicit modeling and some detailed explanations to why implicit representation provides an ideal solution to the modeling of 3D printing ready geometric objects. As will be seen later, implicit modeling is not only a much more natural shape-modeling technique, but more importantly, the models represented by implicit functions are 3D printing ready. In addition to the geometric information, implicit functions can also be used to model complex material structures and material colors, which makes it an ideal 3D object representation for the AM. In “Implicit modeling” section, we will give a brief introduction to a few popularly used implicit modeling techniques, including a recently developed 2D area spline technique. Unlike mesh-based geometric modeling, implicit modeling provides native support to parallel design, which allows to divide a complex geometric design task into a set of smaller and simpler geometric design tasks, which can be processed in parallel simultaneously, owing to the availability of implicit shape-preserving blending operations. The introduction to the shape-preserving operation is provided in “Shape-preserving implicit blending operation” section. In the last part of this paper, we present some key technical challenges related to the development of the AM-oriented CAD technique. Though implicit modeling can also potentially offer the possibility of integrating numerical analysis into implicit function-based CAD design tools [7], the relevant discussions will not be considered in this paper so as to make the paper more focused.

## From explicit modeling to implicit modeling

The direct modeling of an object by an explicit use of points, triangles, or parametric patches is referred to as an explicit method, as one can directly “see” these objects. However, in many ways, explicit surface modeling is not a natural geometric modeling technique. In nature, most objects have volumetric characteristics with highly complex interior structures. In addition, natural objects have an inherently continuous form with infinitely many fine details. This is especially true for biological objects, such as the human bone and vascular structures. As pointed out in [8], natural objects are also often made of hybrid materials and have a hierarchical structure. Another typical feature of natural objects is that they are formed mostly as the result of a procedural process, such as the process when a human body is progressively built up from a single tiny cell. One of the most natural ways of modeling these objects is to emulate the actions or the process through which nature has created these objects. Instead of using points and triangles to specify these objects, in many ways, the process of describing natural shapes by using real functions appears to be more natural and effective, as an implicit function can better reflect the way in which a natural object is being created rather than by using an explicit geometric modeling technique.

**Fig. 1:**
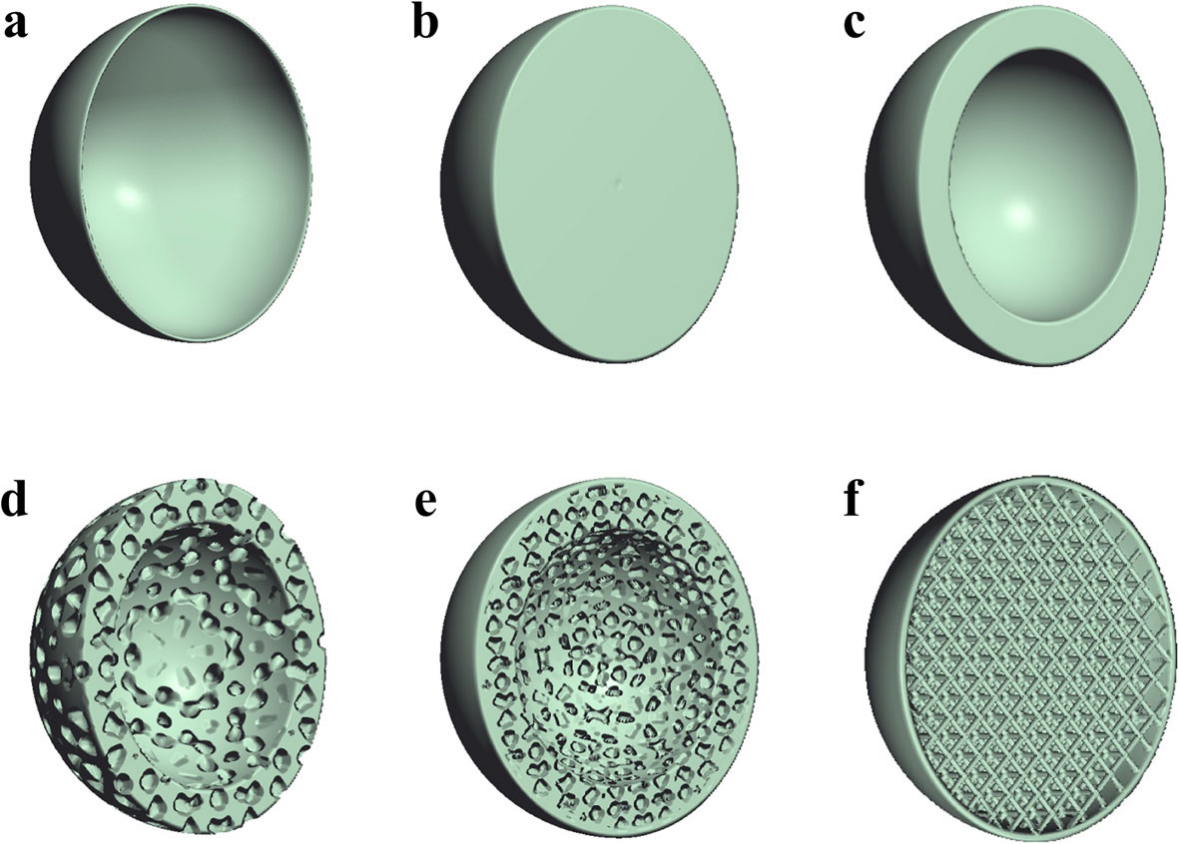
Some simple implicit models to demonstrate the fact that implicitly represented geometric objects are 3D printing ready. These examples show that an implicitly modeled geometric object can not only provide an exterior appearance of an object, but more importantly, it can be associated with a volumetric solid object to provide detailed descriptions about the object’s interior structure and material properties. **a**. Surface representation. **b**. Solid representation. **c**. Solid boundary representation. **d**. Porous material structure. **e** Solid boundary with porous interior materials. **f**. Thin solid surface with interior supporting structures

Implicit modeling has been gaining popularity in recent years in the modeling of visual effects, with the significant increase in the processing power of modern programmable computer graphics hardware. The currently available graphic hardware is not only good at processing explicitly represented geometric objects such as triangle meshes and parametric spline patches, but it can also be programmed and used as a general purpose computing device [9–11]. It is now possible to model and visualize relatively complex objects implicitly in real time and without using any triangle meshes. In general, implicit geometric models are represented by certain kinds of real functions, expressed either in an explicit form or implicitly as an iterative procedure. Granted, a required implicit object can always be created by converting an explicit model into an implicit form, for instance, by means of the distance mapping and by using various implicit fitting techniques. However, such kind of a conversion process can be very time-consuming and computationally expensive, especially when detailed internal geometric structures and material properties need to be considered in the conversion process. This process is simple only when a surface representation is to be converted into a solid. As a matter of fact, converting a boundary-based geometric model into a printable geometric representation is in general a redesign process, if the object is not to be printed directly as a solid. Figure 1 illustrates why this is the case. In this example, a surface-represented sphere just describes the boundary of the object. However, when it is sent to a 3D printer to make the object, though it may be quite straightforward to print it as a solid object, more often it is printed as a hollow sphere to save the printing substance and to improve printing efficiency. In many situations, some supporting structures need to be used inside the object to improve the physical strength of the object. If the internal structure of an object is obtained based on a certain material simulation process, the internal supporting material structure can be quite irregular.

Just as explicit geometries such as triangle meshes are visualization-friendly, implicit geometric representations are by their nature 3D printing-friendly. To understand what makes for a good AM-oriented geometric modeling technique, one has to change their view from the perspective of visualization and subtractive manufacturing to that of the process of building a real object using the AM technique. When an object is used as an input into a 3D printing system, the system must know precisely whether a printing material particle should be laid at a given position. In AM, an object is printed layer-by-layer, and the printing system must know which area of the current printing slice is a part of the geometric model. Thus, in terms of 3D printing, the geometric object should be modeled as a solid rather than as a surface, and an ideal representation of a geometric object can be described mathematically as a mapping

$$F:\mathcal{R}^{3} \to \{0, 1\}, $$

which can be considered as the characteristic function of a 3D point set. When *F*(*x*,*y*,*z*)=1, the point **P**(*x*,*y*,*z*) is on the object, and a tiny printing material particle should be placed at the position. Though a binary-valued function can represent a solid object properly, it lacks the flexibility in terms of the geometric design. Instead of considering a geometric object as a set of points, a general real function $F:\mathcal {R}^{n} \to R$ can be used to specify a geometric object. In fact, any real function can be associated with a surface, a level set of the function *F*(**P**)=0, or a solid object defined by the set of points {**P**:*F*(**P**)≥0} or {**P**:*F*(**P**)≤0}. When an object is represented by a function *F*(*x*,*y*,*z*), the slice corresponding to a level, say *z*=*z*_0_, is just a bivariate function *I*(*x*,*y*)=*F*(*x*,*y*,*z*_0_), which can be used directly as a precise instruction to an AM system to print out the layer. For instance, if *F*(*x*,*y*,*z*) represents the geometry of a digital human body, *I*(*x*,*y*)=*F*(*x*,*y*,*z*_0_) just represents the slice of the human body corresponding to *z*=*z*_0_, similarly to a slice of a 3D medical image. As a matter of fact, any 3D volumetric medical image can be regarded as an implicit function with a gridded discrete domain.

The idea of associating an implicit function with a solid leads directly to one of the most popular implicit shape modeling techniques, known as constructive solid geometry (CSG) [12]. With CSG, the construction of a relatively complex geometric shape can be regarded as a process of combining a set of simple primitive solid objects using set-theoretic operations such as union, intersection, and complement operations. However, the modeling of geometric objects using general implicit functions is much more flexible and powerful than solid modeling. For instance, a non-negative function can be regarded as a kind of an energy function, and a complex geometric shape can be designed as a function corresponding to the total energy generated by a collection of energy sources. Blinn’s popular blob technique [13] can be considered as a typical example developed from this idea. This idea can be generalized through a convolution converting a parametric or a triangle mesh into an implicit object.

When the main application of a geometric model is used for visualization and subtractive manufacturing, explicit geometric modeling methods, such as triangle meshes and parametric geometric surfaces are preferred. However, when a geometric object is modeled for AM, implicitly represented geometric objects are preferred, owing to several distinct advantages of implicit geometric representation over the explicit methods. First, an implicit geometric form is directly defined in the physical space, rather than in the parametric space, and consequently, it can directly provide a 3D printer with precise information about where to lay a printing substance particle. It is a 3D printing ready representation, and no conversion procedure is required. In general, an implicit function can be viewed both as a surface and as a volumetric solid, and it can describe not only the external appearance of an object but also its interior geometric structure and material properties. Second, implicit geometric modeling is a lightweight geometric modeling technique. Unlike geometric objects represented by data-intensive forms such as triangle meshes and point clouds, which often have data of size of over several megabytes, implicitly represented shapes do not in general involve the use of massive data sets, and consequently, do not require a massive storage space. Because of this, implicitly represented geometric objects are also internet and cloud computing-friendly, as it is extremely convenient to transport implicitly represented geometric objects across the internet without any restrictions on the bandwidth of the data transformation over the internet. Another impressive feature of implicit geometric objects is that implicit techniques allow for parallel design, owing to the fact that implicitly modeled objects can be easily combined together [14–17], which makes implicit modeling particularly suitable for the shape design over a distributed or a CAD system with parallel architecture. In addition, implicitly represented geometric models have a collision-detection efficient representation. A 3D object printing operation is in general a process of interaction between a digitally represented geometric object and the printing device, where collision-detection operations have to be constantly performed to test whether a move from the current printing position to the next one is allowed. It is quite straightforward and efficient to perform a collision-detection operation between two objects, when one of them is presented in the implicit form (say, the geometric model) and the other one is in the explicit form (say, the position of 3D printer head).

Before moving to the following sections for more detailed descriptions of some implicit modeling techniques, we illustrate some simple implicit objects in Fig. 1 and show why implicit geometric modeling is 3D printing friendly. As it can be seen later, the 3D forms shown in Fig. 1 can all be easily represented by a simple implicit function, varying from a surface to a volumetric solid with different interior material and supporting structures.

## Implicit modeling

### Implicit modeling using distance functions

The basic principle of implicit modeling can be illustrated directly by using distance functions [18]. For instance, a sphere can be described as a distance function to a point. Similarly, an infinite cylinder can be described as a distance function to a line, and a torus can be described as a distance function to a circle. Several AM techniques based on distance functions have been proposed. In [19], the distance function was used for the boundary voxel optimization. The implicit slicer proposed in [20] is also fundamentally based on the distance function. It was also used by Liu et al. [21] for the design of material composition functions. With the increasing processing power of modern computer systems and the wide recognition of the simplicity and capability of the distance function-based modeling technique, distance functions have recently been popularly used in a variety of applications. For instance, a popular technique known as the Kinect Fusion developed by Newcombe et al. [22] used for the reconstruction of a real-world 3D object from the sensing data is essentially based on the distance function. As the intersection between a ray and a distance function-defined geometric object can be easily calculated by using a numerical method known as ray marching, it has been popularly used for geometric and material modeling in a ray tracing system. For readers who want to know more about the practical use of distance functions, please visit http://www.shadertoy.com, which is a live online visual effect editor in GLSL shader for generating various graphical effects using mainly implicit functions.

### Converting from the explicit representation to the implicit representation

Though distance functions can directly provide the distance information from a given position to the objects that they represent, only a small number of relatively simple objects can be modeled directly as distance functions. As the most popular form of representation of a geometric object, triangle meshes are ubiquitous in the field of computer graphics, 3D games, and CAD. Many complex implicit geometries can be created by converting a triangle mesh model into an implicit representation. One conversion method is to apply the convolution operation to a triangle mesh [23]. Suppose a 3D surface object is represented explicitly by a collection of parametric surface patches, such as triangle meshes $S_{\triangle _{i}}(s,t)$, *i*=1,2,⋯,*N*, $(s,t)\in \mathcal {D}_{i}$. Each point **P**(*x*(*s*,*t*),*y*(*s*,*t*),*z*(*s*,*t*)) on a surface patch is a source of particle energy, and suppose that each particle emits uniformly the same amount of energy defined by a function *K*(*r*)≥0, where $r\in \mathcal {R}$ represents the distance from the surface point **P** to a point *X*(*x*,*y*,*z*) in space. Then, the total energy field generated by the collection of surface patches can be represented by a kind of convolution shown below:

$${}F_{s}(X)= (S\star K)(X) =\sum_{i=1}^{N}\int_{(s, t)\in \mathcal{D}_{i}} K\left(\|S_{\triangle_{i}}(s, t)-X\|\right) dsdt. $$

There are various ways to model the potential function *K*(*r*), but it is often assumed that it is non-negative and decreasing with an increase in distance *r*. Ideally, this function can be described by $e^{-ar^{2}}$ in order to make it more physically meaningful. However, for a potential function defined in this form, it is difficult to find a closed form solution for the convolution defined above. Most often, the following type of function is used [24, 25]:

$$K(r)=\frac{1}{\left(1+ar^{2}\right)^{2}}. $$

**Fig. 2:**
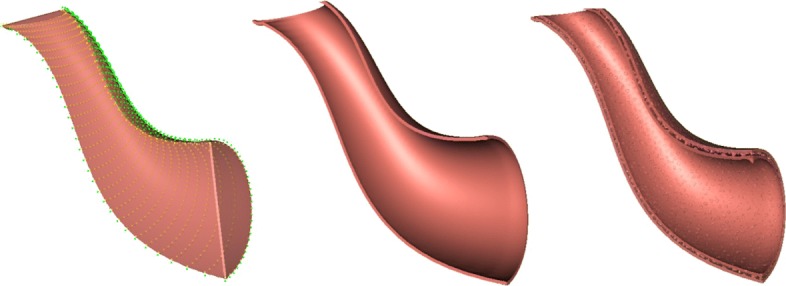
Implicit Utah teapot spout obtained by fitting a point cloud sampled from the classic Utah teapot model. Left: the sampled point cloud; Middle: The implicitly represented thin-solid teapot spout surface; Right: The implicitly represented thin-solid teapot spout with an interior porous material structure

The conversion of an explicit geometric representation to an implicit function can also be achieved by a sampling-fitting process. With this method, a collection of surface points are first sampled from the given explicit form. A certain implicit fitting technique can then be applied to the sampled point cloud to implicitly approximate the given surface [26, 27]. Figure 2 shows the implicitly reconstructed Utah teapot spout by fitting a point cloud sampled from the classic Utah teapot model using the fitting method proposed in [26].

### Procedural implicit modeling

Natural objects are in general a procedural result. Typical examples are the biological objects such as plants, trees, and animals, which build up their geometric forms in a process of cell repetition. The L-system is a powerful technique to model and simulate the process, but it is not a 3D printing friendly representation. The modeling of these objects directly as a real function by simulating the biological growth process in the form of an iterative process seems more natural, especially when the modeling of the internal biological material structures of these objects needs to be taken into consideration.

#### Cell growth simulation

**Fig. 3:**
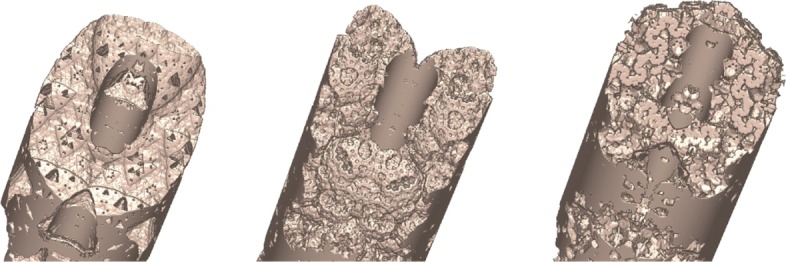
Materials of fractal structures can be easily represented as certain procedural implicit functions

This method is based on the cell growth simulation following the idea of cell division, a process by which a parent cell becomes two or more daughter cells. The process can be modeled by starting from a single cell, which can be initialized as a tiny sphere. This cell then generates new cells, which can be blended together with the older generation of cells and become a relatively bigger cell. In biology, cells behave like stars and planets, which are constantly in motion. By simply combining rotation, translation, and scaling into the simulation process, one can easily model infinitely many different kinds of shapes and material structures (see Fig. 3). One distinctive advantage of the geometries generated in this manner is that they have an infinite level of details, which makes it very suitable for modeling natural objects and biological tissue structures.

#### Procedural non-linear transformations

The idea behind this technique originated from the Mandelbrot set and the Julia set, which can be interpreted as sequences of non-linear geometric transformations. Indeed, for a complex number *z*=*x*+*yi*, *z*^2^+*C* actually represents a combination of rotation, scaling, and translation operations. This becomes obvious when we rewrite *z* as *z*=*re*^*iθ*^, where $r=\sqrt {x^{2}+y^{2}}$, $\theta =arctan\frac {y}{x}$. In fact, *z*^2^=*e*^*iθ*^×*z* actually corresponds to a rotation of the point positioned at (*x*,*y*) by an angle *θ* around the coordinate origin. The generalization of the 2D Mandelbrot set or the Julia set to 3D is usually done with the quaternion *q*=*xi*+*yj*+*zk*+*w*, but it is difficult to generate meaningful geometric objects by formulating the iterative process by using the formula $q_{1}=q_{0}^{2}+C$, as a quaternion is in general a 4D object, which can only be visualized in 3D slice-by-slice. Recently, some effort has been made by following a geometric intuition, such as by using the famous MandelBulb 3D fractal object defined by Daniel White [28].

In general, this idea can be generalized in the following way. For any point **P**(*x*,*y*,*z*), let **P**_0_=(*x*,*y*,*z*) and

$$\mathbf{P}_{n+1}=\left(\mathcal{X}(\mathbf{P}_{n}), \mathcal{Y}(\mathbf{P}_{n}), \mathcal{Z}(\mathbf{P}_{n})\right) + T, \hskip 0.5cm n=0, \cdots, N. $$

where $\mathcal {X}(x, y, z), \mathcal {Y}(x, y, z), and \mathcal {Z}(x, y, z)$ are three implicit functions and *T* is a fixed 3D translation. Apparently, various fractal forms can be defined in this way. However, how to define a proper transformation to generate a required form is largely a trial and error process.

### Implicit modeling using Li-Tian’s area splines

**Fig. 4:**
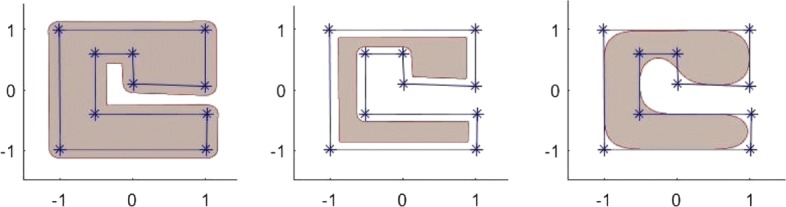
The left two figures show the areas defined by the distance function to the polygon boundary. The right figure shows the area defined by the implicit spline functions built from the given polygon. As it can be seen from the figure, the solid area modeled by using the 2D implicit spline technique behaves more like traditional parametric splines curves

One challenging problem in implicit modeling is that it lacks a technique similar to various explicit spline techniques for modeling free-form implicit objects. While the popular blob-based technique is very effective in the modeling of soft deformable objects, it is difficult to model free-form implicit geometric objects. One way of modeling free-form implicit objects has been the application of the distance functions to a polygon or a polyhedron specified by some control points. However, there are some drawbacks to this method. One difficulty is the integration in the distance function of both the flexibility of specifying the smoothness of a required free-form shape and the accuracy of the shape approximation to the control polygon, which is one of the most important features of parametric spline techniques. To achieve a high level of smoothness around the vertices, when using the distance function to a polygon, a relatively larger value of the distance function has to be used, which will subsequently result in a poor approximation of the original geometric shape specified by the base polygon, as it can be seen in the left two figures shown in Fig. 4.

**Fig. 5:**
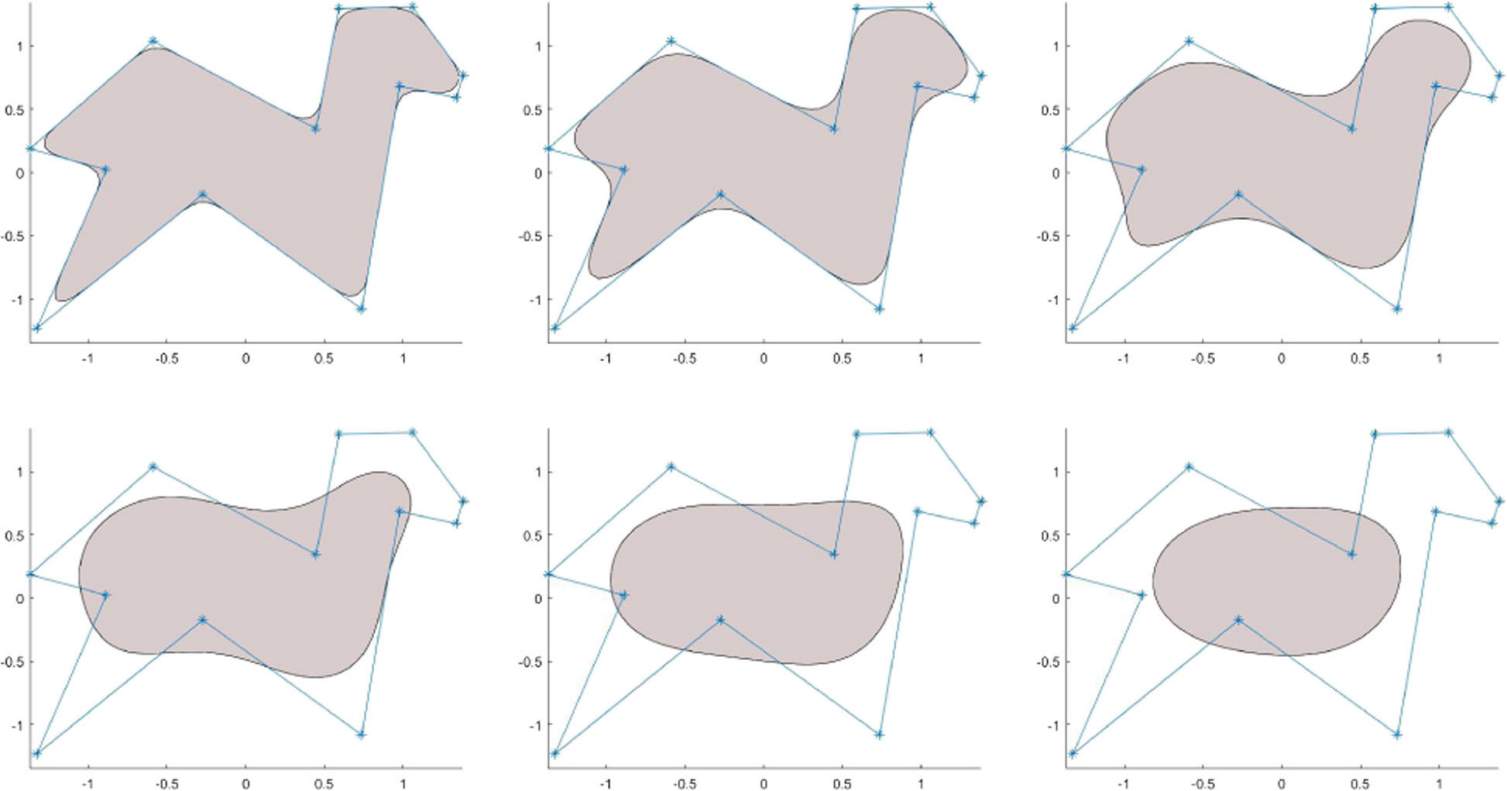
The shapes of these areas are defined by the *C*^2^-smooth implicit function $B_{\Delta,\delta }^{(3)}(x,y)$ corresponding to the level value of less than 0.5. The underlying control polygon for constructing $B_{\Delta,\delta }^{(3)}(x,y)$ is specified using the same set of vertices **P**_0_,**P**_1_,⋯,**P**_8_. All these shapes differ only by the *δ* values, which are *δ*=0.05,*δ*=0.15,*δ*=0.25,⋯,*δ*=0.55, respectively

Another way of constructing a free-form implicit shape is to specify an object as a point cloud and construct an implicit function from the given point set based on a certain surface approximation and interpolation technique [26, 29–31]. However, most of these kinds of techniques are in general computationally expensive and involve the use of a massive 3D data set, which often leads to poor performance, if the data set is very big. Recently, a kind of an implicit free-form shape modeling technique has been developed by Li & Tian [32], which can be used to design implicit objects in a similar way to the conventional parametric spline shapes (See Fig. 5).

The basic idea of the implicit spline is to sub-divide a 2D region into a collection of polygons with different potential functions defined on different regions. Similarly to the control points-based parametric spline techniques, implicit spline objects can be designed as a convex blending of a set of implicit potential field functions corresponding to different regions. These locally defined field functions behave similarly to the control points used in a traditional spline technique and can be referred to as control implicit primitives. The main difference between our 2D implicit splines and an explicit spline technique is that the shape defined by the 2D implicit spline technique corresponds to a solid area, whereas the shape defined by a conventional spline is only a boundary.

The key challenge in our technique is how to construct the basis functions corresponding to a given set of 2D polygons with each individual polygon having an arbitrary shape. Similar to conventional spline basis functions, it is generally expected that the basis functions built from the set of polygons are polynomial, non-negative, and have the property of the partition of unity, if the initial polygons form a partition of a 2D domain. Apparently, polygon-based distance functions do not meet these basic requirements. Our way of constructing the required bivariate spline basis functions is to find a general solution to the following integral convolution, which is similar to the construction of conventional B-spline basis functions. Let $\square \subset \mathcal {R}^{2}$ be a square of size 2*δ*×2*δ* centered at the coordinate origin with *δ*>0. For an arbitrarily given polygon $\Delta \subset \mathcal {R}^{2}$, we define a sequence of functions in the following way:

$$ {{\begin{aligned} B_{\Delta,\delta}^{(0)}(x, y)\! &\,=\, \chi_{\Delta} (x,y)=\left\{ \begin{array}{ll} 1, & \text{(x,y)} \in \Delta; \\ 0, & \text{(x,y)} \notin \Delta. \end{array}\right. {,}\\ B_{\Delta,\delta}^{(n)}(x,y) \!&\!= \frac1{4\delta^{2}} \!\int\int_{\mathcal{R}^{2}} \!B_{\Delta,\delta}^{(n-1)}(s,t) \chi_{\square}(s\,-\,x, t\,-\,y)dsdt, \,\,\, \,\,2cm(n\!>\!\!0). \end{aligned}}} $$

The parameter *δ* in the integral serves as a solid polygon vertex smoothing parameter, which specifies the extent to which one wants to smooth a sharp vertex corner of a polygon. With the properties of integration, it can be seen clearly that each $B_{\Delta,\delta }^{(n)}(x,y)$ defined in this way has the following two properties. First, $B_{\Delta,\delta }^{(n)}(x,y)$ is a piecewise polynomial function. Second, $B_{\Delta,\delta }^{(n)}(x,y)$ is *C*^*n*−1^ continuous. Though this idea of constructing the required spline basis functions is simple, without an explicit expression of these convolutions, the numerical evaluation of these functions can be very expensive. Fortunately, we have found a way to express these convolutions explicitly in the analytical form.

As it has been shown in [32], the function defined above can be expressed explicitly as a linear combination of a set of bivariate functions $\Omega ^{(n)}_{E, \delta }(x,y)$ associated with different edges of polygonal *Δ* and the piecewise polynomial smooth unit step functions *H*_*n*_(*x*). For a polygonal edge parallel to the 2D vector *E*(*α*,*β*),*α*,*β*>0, $\Omega ^{(n)}_{E, \delta }(x,y)$ can be expressed as

1 $$ \begin{aligned} \Omega^{(n)}_{E, \delta}(x,y)\,=\,\frac 1 {(4\delta^{2})^{n}}\! \sum^{n}_{i=0}\sum^{n}_{j=0}(-1)^{i+j} C_{n}^{i}C_{n}^{j} A_{E}^{(n)}\\ \times (x\,+\,(n\,-\,2i)\delta, \, y\,-\,(n\,-\,2j)\delta), \end{aligned}  $$

where

2 $$ {{\begin{aligned} {}A^{(n)}_{E} (x,y)\,=\,\left\{ \begin{array}{ll} 0, & \quad \!\!\alpha y\ge \beta x \,or \,y\ge 0;\\ \!\frac{1}{(2n)!\alpha^{n}\beta^{n}} (\beta x - \alpha y)^{2n}, & \quad \!\!\alpha y< \beta x, y<0, x\!\le\! 0;\\ \sum_{k=1}^{n}\frac{(-1)^{n+k}\alpha^{k}}{(n-k)!(n+k)!\beta^{k}}x^{n-k}y^{n+k}, & \quad \!\!\alpha y< \beta x, y<0, x\!>\! 0; \end{array}\right. \end{aligned}}}  $$

and the piecewise polynomial smooth unit step function *H*_*n*_(*x*) is defined recursively by

$$ {{\begin{aligned} H_{0}(x)&=\left\{ \begin{array}{ll} 0, & \quad x<0; \\ \frac12, & \quad x=0; \\ 1, & \quad x>0. \\ \end{array} \right.\\ {}H_{n}(x)&= \frac12 \left(\!\left(\!1\,+\,\frac {x} {n}\right)\! H_{n-1}\!(x\,+\,1) \,+\, \left(\!1\,-\,\frac {x} {n}\right)\!H_{n-1}\!(x\,-\,\!1)\!\right)\!, \!n\,=\,1, \!2, \!3, \cdots. \end{aligned}}} $$

Note that when the value of the polygon smoothing parameter *δ* is sufficiently small with respect to the size of the polygon, we have $B_{\Delta,\delta }^{(n)}(x,y)=1$ for the most part of the interior region of the given polygon. Thus, when $B_{\Delta,\delta }^{(n)}(x,y)$ is used as a weight function combining a control implicit primitive function *F*(*x*,*y*), we have $B_{\Delta,\delta }^{(n)}(x,y)F(x,y)=F(x,y)$, when $B_{\Delta,\delta }^{(n)}(x,y)=1$. When associating *F*(*x*,*y*) with a 2D implicit geometry, the new function $B_{\Delta,\delta }^{(n)}(x,y)F(x,y)$ will have exactly the same shape as that defined by *F*(*x*,*y*), when the point **P**(*x*,*y*) is well within the support of $B_{\Delta,\delta }^{(n)}(x,y)$, while the part of the shape defined by *F*(*x*,*y*) that is well outside the support of $B_{\Delta,\delta }^{(n)}(x,y)$ is removed as $B_{\Delta,\delta }^{(n)}(x,y)F(x,y)$ will be nearly zero. Therefore, the function $B_{\Delta,\delta }^{(n)}(x,y)$ can be referred to as a kind of a shape-preserving spline basis function.

It can be shown directly that bivariate functions $B_{\Delta,\delta }^{(n)}(x,y)$, *n*=0,1,2,⋯, have the following properties:

1. *Nonnegativity:*$0\le B_{\Delta,\delta }^{(n)}(x,y)\le 1$.
2. *Smoothness:*$B_{\Delta,\delta }^{(n)}(x,y)$ has a *C*^*n*−1^ continuity.
3. *Piecewise Polynomial:*$B_{\Delta,\delta }^{(n)}(x,y)$ is piecewise polynomial.
4. *Local Support:*$B_{\Delta,\delta }^{(n)}(x,y)$ has a finite support if *Δ* is finite.
5. *Additivity:*$B_{\Delta,\delta }^{(n)}(x,y)$ is additive. That is, if two polygons *Δ*_1_ and *Δ*_2_ do not intersect or they only intersect at their edges, then
  $$B_{\Delta_{1}\cup\Delta_{2},\delta}^{(n)}(x,y)= B_{\Delta_{1},\delta}^{(n)}(x,y)+B_{\Delta_{2},\delta}^{(n)}(x,y). $$
6. *Partition of unity:*$B_{\Delta,\delta }^{(n)}(x,y)$ takes value in [0,1] and if
  $$\bigcup_{k}\Delta_{k} = \mathcal{R}^{2}, \hskip 0.1in area\left(\Delta_{i}\bigcap_{i\ne j}\Delta_{j}\right)=0, $$
  then
  $$\sum_{k} B_{\Delta_{k},\delta}^{(n)}(x,y)= 1. $$

The solid areas shown in Fig. 5 show the filled contour $\left \{(x,y): B_{\Delta,\delta }^{(3)}(x,y)\le 0.5\right \}$ corresponding to different *δ* values for the spline basis function built from the same control polygon *Δ*. As it can be seen from the figure, the shape of the control polygon can be approximated at a varying level by using a single parameter *δ*: the smaller the *δ* value, the more closely the filled contour of the spline basis function approximates the control polygon.

**Fig. 6:**
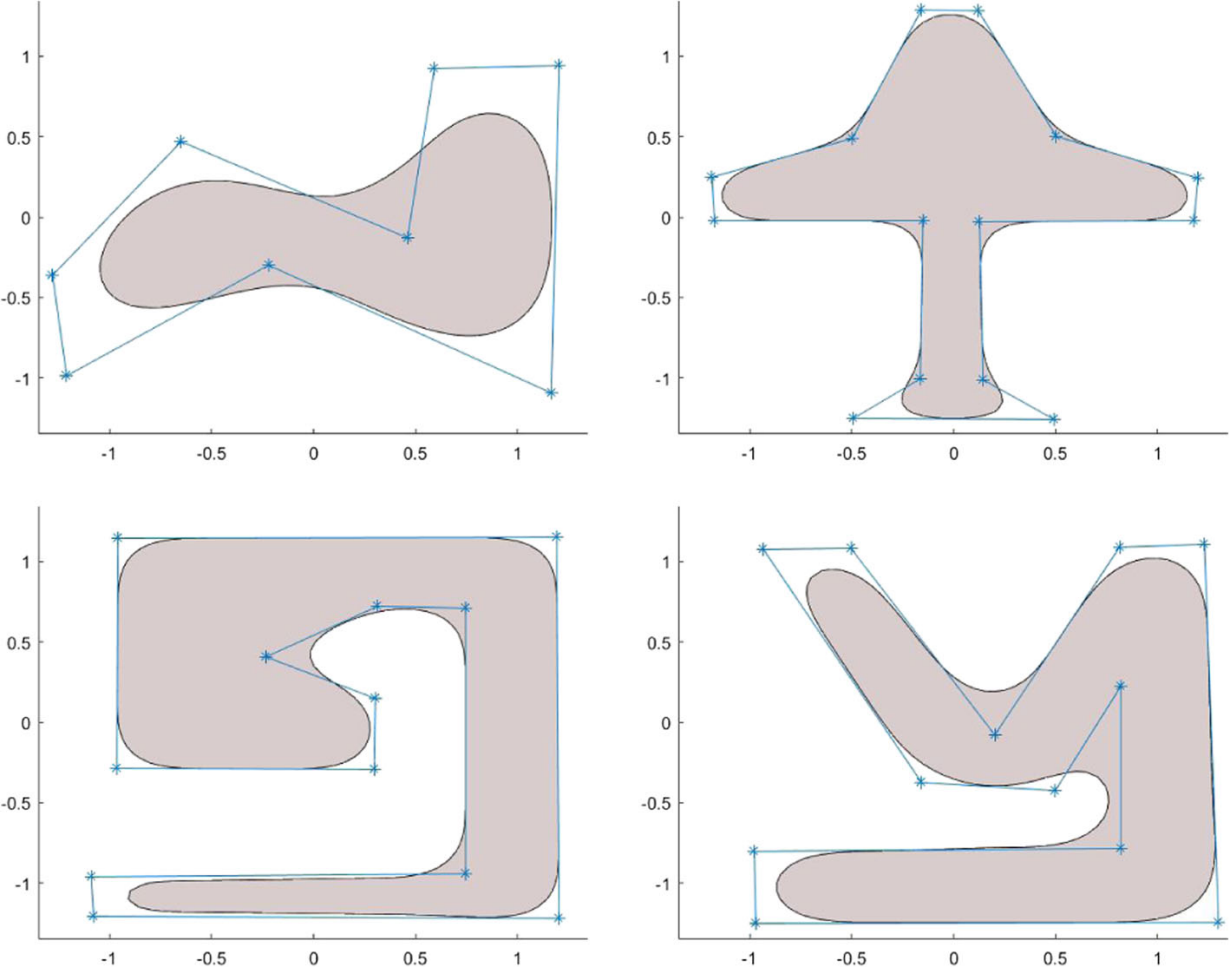
Free-form implicit 2D solid shapes designed based on some simple polygons. All the underlying implicit functions $B_{\Delta,\delta }^{(n)}(x,y)$ are *C*^2^ continuous

The design process of the free-form solid area spline is similar to the design process of spline curves using a conventional spline technique. In fact, to find $B_{\Delta,\delta }^{(n)}(x,y)$ for a given polygon *Δ*, one needs only to specify the control points in the counter-clockwise order and to choose a proper degree of smoothness of the required bivariate function as well as the polygon smooth parameter *δ*. Some more 2D implicit shape design examples are shown in Fig. 6.

Figure 4 illustrates the difference between our 2D implicit spline technique and the distance functions. Distance functions have gained considerable attention recently, given that they support the fast ray marching of distance function-defined implicit objects. However, distance functions are not good at modeling free-form objects. Though the feature presented by our implicit spline can be achieved by using a distance function defined by a set of connected piecewise low-degree polynomials, it is difficult to achieve a high level of smoothness, such as *C*^2^-smoothness. As described above, when a big polygon is subdivided into a set of smaller sub-polygons, the implicit function built from the big polygon is exactly the sum of all the basis functions constructed from the set of smaller polygons. When the distance function is used for a free-form solid area design, the distance function corresponding to the big polygon is the minimum of all distance functions of the sub-polygons, and consequently, the property of the partition of unity is not preserved.

With the proposed bivariate splines, a free-form implicit function *f*(*x*,*y*) can be generated intuitively by laying out a sequence of control points or a sequence of control implicit primitives, similarly to the way one models a shape using, say, B-splines. Suppose **P**_*k*_(*x*,*y*), *k*=0,1,⋯,*m* are the locally specified implicit functions with their main features defined on polygons *Δ*_*k*_, *k*=0,1,⋯,*m*, respectively. Then, these *m*+1 implicit functions can be combined as a weighted sum of the *m*+1 implicit polygons and described in the following way:

3 $$ F(x,y)=\sum\limits_{k=0}^{m} \mathbf{P}_{k}(x,y)B_{\Delta_{k},\delta}^{(n)}(x,y),  $$

where $B_{\Delta _{k},\delta }^{(n)}(x,y)$ is the implicit spline basis function constructed from the polygon *Δ*_*k*_.

The representation of a binary implicit function as a sum of weighted implicit spline basis functions can have various applications. As described in [33], it can be used directly to simplify the task of fitting a big 3D point cloud captured by a modern depth camera. The shape-preserving feature of the proposed implicit spline basis functions allows to sub-divide the depth map captured by a camera into smaller regions. A shape-fitting technique can then be used to fit each sub-dataset individually and then combine them together. The shape-preserving feature guarantees that the main features of each individually fitted shape are maintained, when they are combined together, based on the equation shown in (3). This idea can be very useful, as a complex surface-fitting task can be divided into a set of simpler fitting tasks and implemented directly in a parallel processing system.

The idea can also be applied to a complex implicit geometric design. The shape-preserving feature of the proposed spline basis functions allows us to simplify a complex geometry design task into a process of designing a set of simpler geometric components. The shape-preserving feature of basis functions is becoming more essential when parts of a designed 2D region are taken directly from a slice of a real-world 3D object.

**Fig. 7:**
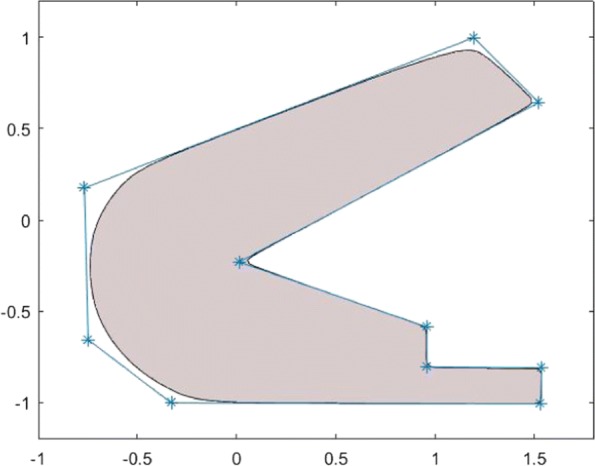
A free-form 2D implicit area corresponding to a given polygon can be obtained by smoothing different vertices differently by using different smoothing parameter values

According to the way in which each individual implicit spline basis function is defined, the free-form solid area corresponding to a polygon is obtained by smoothing each vertex of the polygon by using a uniform smooth parameter value. In practice, one might want to apply different vertices with different smoothing parameter values to enhance the flexibility and the capability of the design technique. Since implicit shapes can be easily combined together in a set of solids by using set operations, this objective can be achieved easily by using implicit function-blending operations. Another way to achieve this design feature is to subdivide the given polygon into a set of smaller polygons and specify different smoothing parameter values for different sub-polygons. In this way, different sets of implicit spline basis functions are created by using different values of the smoothing parameter *δ*. These implicit functions can then be combined, following the idea illustrated in Eq. (3). The 2D implicit shape shown in Fig. 7 is obtained in this way.

### 3D implicit geometric design using 2D implicit functions

In addition to the above-mentioned direct applications of the area spline technique, 2D area splines can also be used in a number of different ways to design 3D implicit shapes. Just as a surface can be regarded as a family of curves, a volumetric solid object can be regarded as a family of 2D slices, or the volumetric region is swept by moving a 3D solid object or a slice of a 3D object. This idea leads to a number of ways in which the 3D implicit object design can use 2D implicit functions.

#### Implicit shape of extrusion

The creation of explicit geometric surfaces by extruding a parametric curve is a powerful and popular geometric design technique. This idea can also be followed when creating 3D implicit objects by using 2D implicit functions [16, 34]. One simple example is the implicit description of a cylinder. As it is commonly known, a cylinder can be described as a distance function to a line. However, it can also be described as an extrusion of a solid disc along a line. Suppose the line is defined implicitly as the intersection of two orthogonally oriented planes *π*_1_(*x*,*y*,*z*)=0 and *π*_2_(*x*,*y*,*z*)=0. Let *C*(*x*,*y*)=*x*^2^+*y*^2^ be the binary implicit function corresponding to the cross-section of a cylinder. Then, the composite function

$$F(x, y,z)=C(\pi_{1}(x, y,z), \pi_{2}(x,y, z)) $$

corresponds to the implicit function of the cylinder with its central line defined by planes *π*_1_(*x*,*y*,*z*)=0 and *π*_2_(*x*,*y*,*z*)=0.

This idea can be immediately generalized to the description of other more general geometric objects. Suppose an extrusion path is represented implicitly by the intersection of two distance surfaces *F*_1_(*x*,*y*,*z*)=0 and *F*_2_(*x*,*y*,*z*)=0, such that they intersect orthogonally. Let the cross-sectional profile curve be defined as an implicit function *C*(*x*,*y*)=0. Then, an extruded implicit object can be directly described by

$$C(F_{1}(x,y,z), F_{2}(x,y,z))=0. $$

**Fig. 8:**
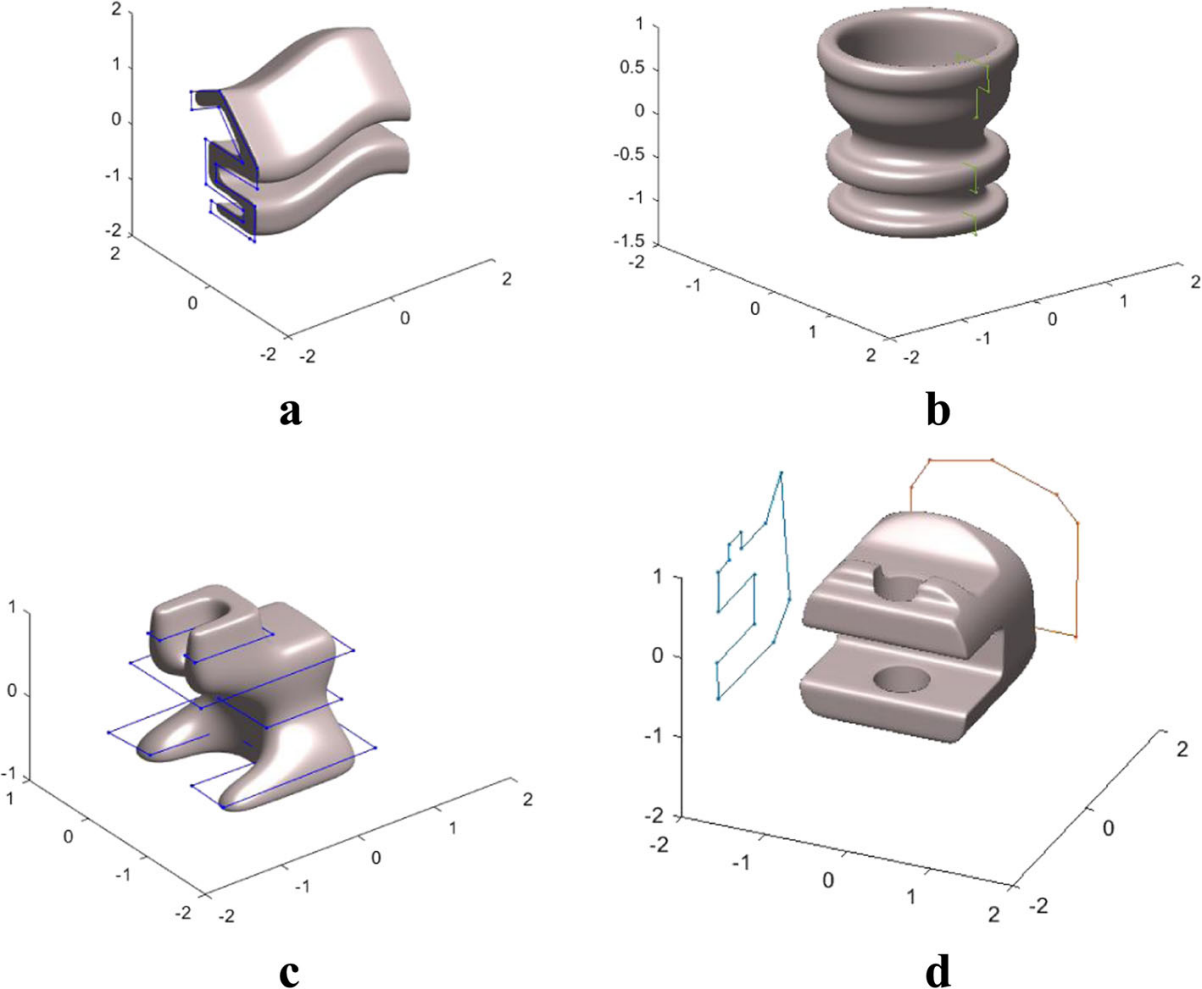
3D implicit object design using the 2D implicit functions: **a**. Implicit geometric object of extrusion. **b**. Implicit geometric object of revolution. **c**. Implicit geometric object design based on a stack of parallel 2D implicit slices defined by bivariate functions. **d**. Implicit geometric object design based on silhouette profiles

However, the specification of the extrusion path as the orthogonal intersection of two distance function-defined surfaces is a practically challenging task. This is because the type of implicit surfaces that can be defined by distance functions is very limited. In addition, except for a few simple implicit functions, it is very difficult in general to check whether two given implicit surfaces are orthogonally intersected. To make the above implicit design method more flexible, the two implicit functions for the specification of the extrusion path can be replaced by two general signed implicit functions. However, it should be noted that the cross-sections of the extruded 3D implicit shape may not necessarily be identical, when the two implicit functions used to define the extrusion path are not the distance functions or when they are not orthogonal along their intersection. A simple implicit object generated by an extrusion is shown in Fig. 8a.

The idea of creating the 3D implicit geometry by extruding a solid 2D implicit profile can be directly implemented by simulating the process, when a sculptor creates a piece of sculpture. In this case, the volumetric region swept by a sculpting knife can be modeled as an extrusion of an implicit function along the path of the motion of the sculpting knife, and the cut of an implicitly represented sculpture can be represented directly as a result of an implicit blending of the two implicit objects.

#### Implicit shape of revolution

The creation of a geometric object by rotating a given 2D profile is also very popular in geometric design. This idea can also be introduced in the creation of the implicit geometry of revolution. In fact, the implicit revolution can be considered as a special case of implicit extrusion, where the extrusion path is defined by an implicit cylinder and a plane. Suppose the revolving profile of a 2D implicit object is represented by a function *F*(*x*,*y*). Then, the implicit geometry of revolution generated by rotating the implicitly represented profile about the y-axis can be described by $F\left (r-\sqrt {x^{2}+z^{2}},y\right)=0$, and the implicit geometry of revolution generated by rotating the implicitly represented profile about the x-axis can be described by $F\left.\left (x, r-\sqrt {y^{2}+z^{2}}\right)\right)=0$. Fig. 8b shows an example of an implicit geometry of revolution obtained by rotating an implicit spline about the z-axis.

#### Implicit shape as a set of control profile functions

The design and reconstruction of 3D shapes based on planar cross-sections has long being recognized as an effective way of the shape-modeling technique [35–38]. This technique is especially useful in the reconstruction of human organs, such as lungs, heart, and vascular systems [39, 40].

Just as 3D parametric spline surfaces can be considered as a blending of a set of cross-sectional profile curves, any free-form implicit shape can be designed as a blending of a set of 2D implicit shapes, which serve as local control profiles, with each of these 2D implicit functions specifying a cross-sectional profile of a required solid shape. One simple and direct method is to specify a required solid shape as a set of slices along a coordinate axis, say, the z-axis, and to represent the overall geometric shape of the object by using a certain spline technique. That is, we can describe a required implicit 3D object in the following form:

4 $$ F(x, y, z)=\sum_{k=0}^{m} S_{k}(x,y)B_{k}(z)=0,  $$

where $\{B_{k}(z)\}_{k=0}^{m}$ are a certain type of spline basis functions. Shapes presented in Fig. 8c are generated in this manner using *C*^2^-smooth spline basis functions and by using the Bezier spline basis functions.

#### Silhouette based implicit modeling

**Fig. 9:**
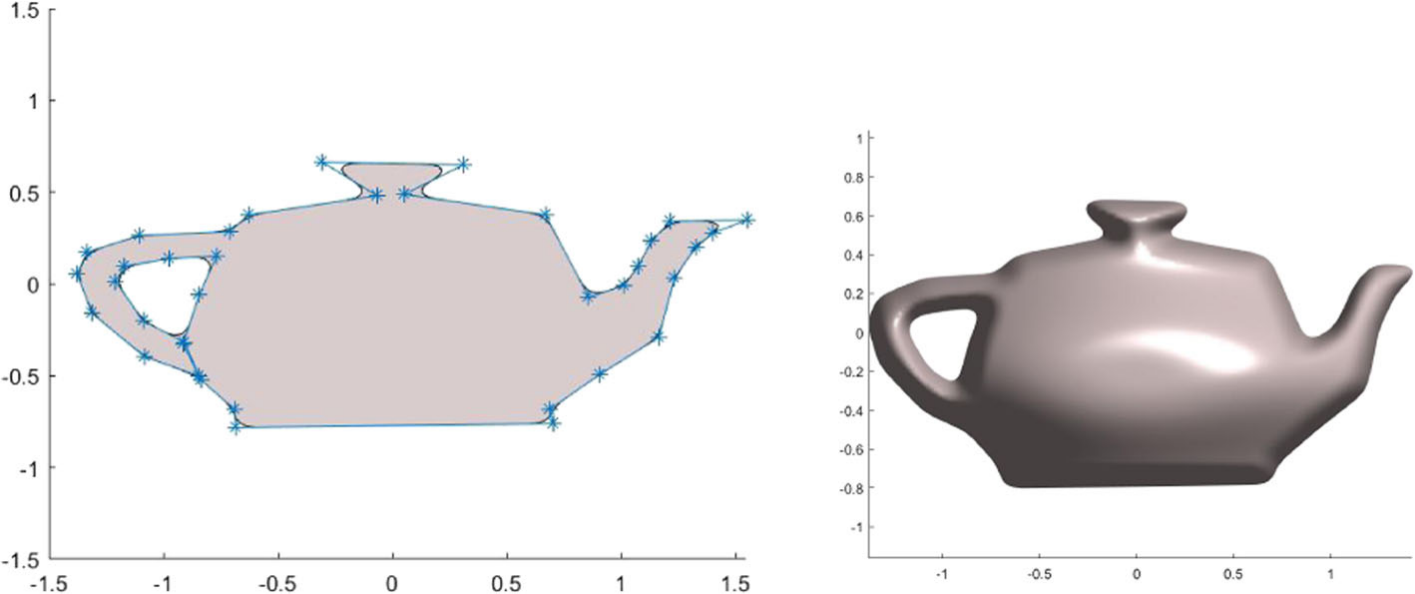
2D area splines are also useful in drawing-based 3D interactive modeling. This figure illustrates how to quickly create 3D models by using a 2D silhouette profile and depth functions

Area splines are also useful for the implementation of 2D drawing-based modeling. Silhouette profiles are an effective feature in the modeling of 3D objects [41]. Figure 8d demonstrates how an implicit object can be designed in this way. This 3D object design method is not only natural in terms of the human vision but is also very effective. However, when modeling a relatively complex object, a large number of profiles is required, which can be quite computationally expensive. A much more effective design method is, when a view is specified, to specify not only the silhouette profile of a required shape but also the depth information, which can be described also as a 2D implicit function *depth*=*D*(*x*,*y*) in the view space. Figure 9 illustrates how it works, by combining both the silhouette profile and the depth information, where the silhouette profile is described by using Li-Tian’s implicit spline technique. A set of these view-space-specific implicit forms can be transformed into the world space and combined together to form a complete description of a required geometric object by using a certain shape-preserving implicit blending operation, which will be addressed in “Shape-preserving implicit blending operation” section.

### Volumetric material structural modeling

The implicit geometric modeling method is also very flexible and effective in the modeling of real-world volumetric forms, varying from fabric objects design to biological tissues and human vascular and neural systems. Due to the high diversity of natural forms, it is impossible to show case by case how each of them can be described by using an implicit function. Here, we illustrate the potential and the flexibility of implicit modeling by using two simple examples.

**Fig. 10:**
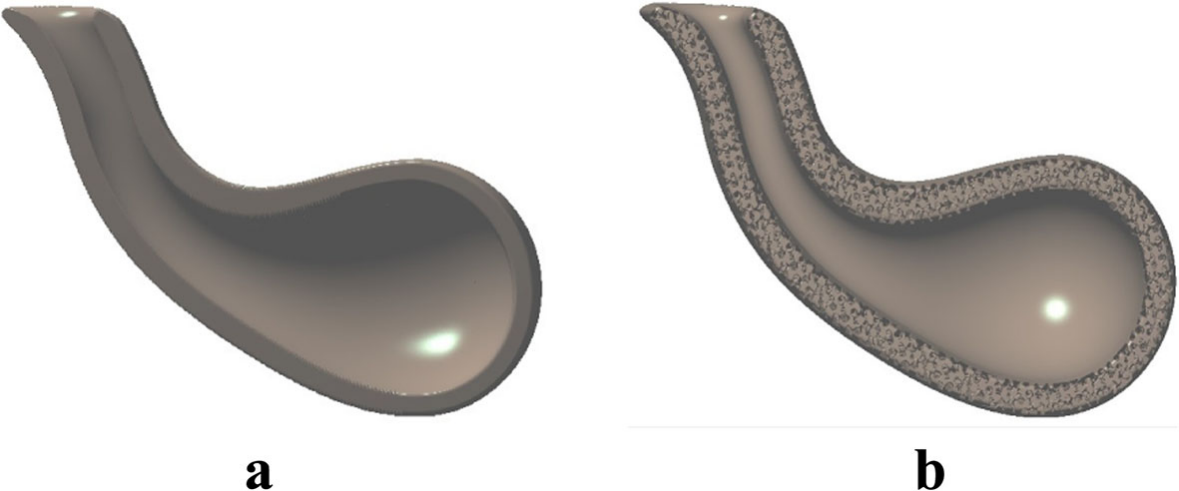
**a**. Implicitly represented solid teapot spout. **b**. The external geometric appearance of the teapot spout and its internal material structure is combined directly as a blending of implicit functions

**Fig. 11:**
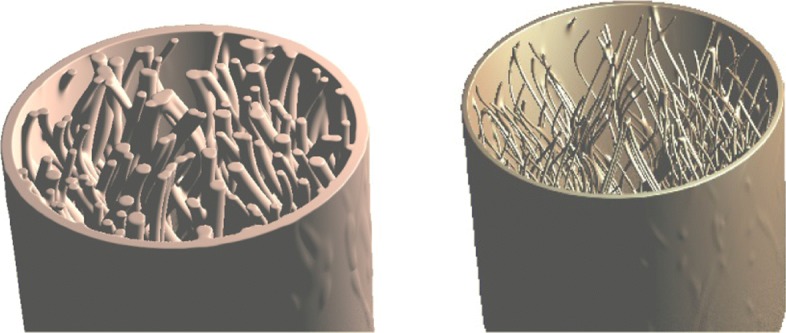
The volumetric nature of biological tissue structures can be represented directly as the blending of an implicitly represented solid shape and its interior tissue structures

As shown in Figs. 10 and 11, the external look of an object and its internal material structure can both be modeled implicitly and combined together as a blending of real functions.

**Fig. 12:**
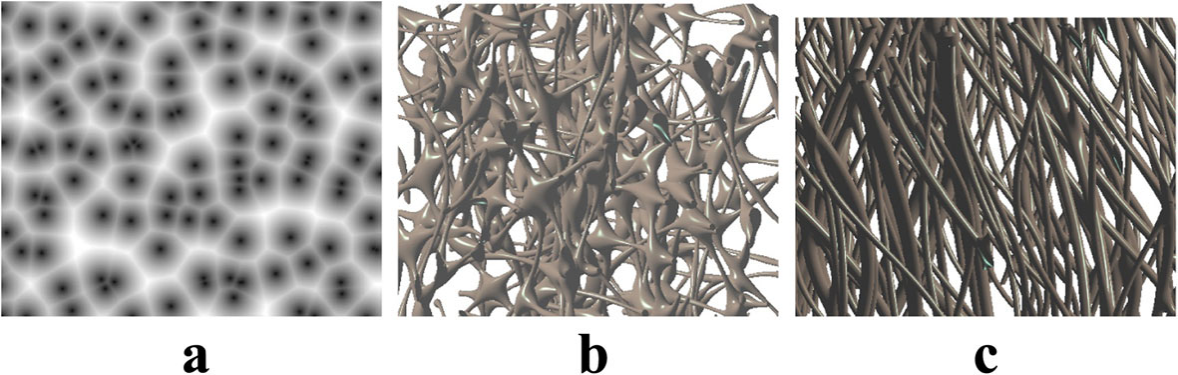
Implicit geometric modeling of the biological neural system (**b**) and the vascular cluster (**c**) using some 2D distance functions (**a**)

Figure 12 shows how a highly complex neural system and a cluster of micro blood vessels can be described implicitly by simply using a few 2D implicit functions. The two 3D implicit objects (Fig. 12b and c) are all modeled by combining a few 2D distance functions, each of which corresponds to the distance to a given set of 2D positions (Fig. 12a).

## Shape-preserving implicit blending operation

One of the most significant features of implicit modeling is that different individually modeled shapes can be combined easily by using some very simple implicit shape-blending functions. In general, any binary function *O*(*x*,*y*) can be used to combine two implicit functions. Let *F*_1_(*X*), *F*_2_(*X*) be the implicit functions corresponding to the shapes *A*,*B*. Then, the compound function *O*(*F*_1_(*X*),*F*_2_(*X*)) defines a new implicit function, whose corresponding geometric shape can be regarded as the combination of the shape *A* and the shape *B*. For instance, when an implicitly defined geometric shape is regarded as a solid, the binary functions *O*(*x*,*y*) corresponding to point-set theoretical operations like union, intersection, and subtraction operations can be defined directly by using max(*x*,*y*) or min(*x*,*y*). However, geometric shapes obtained from such simple binary operations are in general not smooth at the joint. To achieve smooth blending at the joint of two implicit shapes, a certain smooth blending operation has to be used. Some basic requirements to define a “good” blending operation have been proposed in [42, 43]. Even though in general there are no commonly accepted criteria about exactly whether a blending operation is “good”, it is generally agreed on that a good blending operation should not only be able to generate smooth implicit shapes, but can also be performed in a controllable way. To be more specific, we expect a blending operation to possess a kind of a shape-preserving property. To smoothly combine two implicit shapes, some local deformation of the original shapes is inevitable, but we want the local deformation to apply only to the regions close to where the two geometric shapes intersect. The shape-preserving feature of an implicit blending operation is of essential importance. This is because, with the availability of shape-preserving blending operations, a complicated task of designing a relatively complex geometric object can be sub-divided into a set of simple geometric object-design tasks. So far, several smooth shape-preserving blending operations have been proposed. In [42], smooth-blending range-controllable operations were defined by using a scalar function. In [43], the R-function was used to achieve the blending features. Smooth shape-preserving Boolean operators were also introduced in the work of Barthe et al. [44]. The major limitation of all these blending operations is that they only have the *C*^1^ or the *G*^1^ continuity and lack simplicity in their geometric representations. Comparatively, the piecewise polynomial-blending operations (PPBO) proposed in [15] have several advantages. PPBO are not only shape-preserving, but they can also be defined directly to have any required degree of smoothness. In addition, they are defined as piecewise polynomials and have a simple form in their mathematical expressions. A brief survey of various blending operations can be found in [45], though there is a lack of a sufficient review of shape-preserving blending operations. Owing to the importance of the shape-preserving feature of a blending operation in implicit modeling, here we give a brief introduction to PPBO.

### **Definition 1**

Let $|x|: \mathbb {R}\rightarrow \mathbb {R}$ be the conventional absolution function. That is, |*x*|=*x* when *x*≥0 and |*x*|=−*x* when *x*<0. Then we introduce the following generalized absolute functions:

5 $$ \begin{aligned} |x|_{0} & =|x|;\\ |x|_{n}& = \frac1{2(n+1)}((n\,-\,x)|1\,-\,x|_{n-1} \,+\, (n+x) |1+x|_{n-1}).\\ n &=1, 2, 3, \cdots \end{aligned}  $$

|*x*|_*n*_ is called the degree *n* of the absolute function.

It can be shown that |*x*|_*n*_ has the following properties:

1. |*x*|_*n*_≥|*x*|, and |*x*|_*n*_=|*x*| when |*x*|≥*n*;
2. |*x*|_*n*_ is *C*^*n*^-continuous;
3. |*x*|_*n*_ is a piecewise polynomial function.

From this definition, we can immediately write out the *C*^2^−smooth absolute function as

$$|x|_{2}=\left\{ \begin{array}{ll} |x|, & |x|>2; \\ \frac{x^{2}}{2}\left(1 - \frac{1}{6}|x|\right) +\frac23, & |x| \le 2; \end{array}\right. $$

The recursive definition of the degree *n* of the smooth absolute function shown in Definition 1 is actually obtained in the following way as the function convolution,

6 $$ |x|_{n}=\frac12\int_{x-1}^{x+1}|t|_{n-1}dt=\int_{-\infty}^{\infty}g(x-t)|t|_{n-1}dt,  $$

where

$$g(t)= \left\{ \begin{array}{ll} \frac 1 2, & \quad t\in [-1, 1]; \\ 0, & \quad \text{otherewise.} \\ \end{array} \right. $$

An interesting thing about the integration (6) is that not only it can be evaluated in a recursive way, but it can also be written explicitly in the following form:

$$\begin{array}{@{}rcl@{}} |x|_{n} = \frac1{(n\,+\,1)! 2^{n}}\sum_{k=0}^{n-1}(-1)^{k} C_{n-1}^{k}G_{n}(x+ n - 2k-\!1), \, n=1, 2, 3, \cdots \end{array} $$

where the function *G*_*n*_(*x*) is defined as

7 $$\begin{array}{@{}rcl@{}} {}G_{n}(x) \,=\, (x\,+\,1)^{n}|x\,+\,1| \,-\, (x-1)^{n}|x\,-\,1|, \quad \!\!n\,=\,1, 2,3, \cdots \end{array} $$

For example, with the Eq. 7, we can also write out the *C*^2^− smooth absolute function |*x*|_2_ immediately as

$$x|_{2}=\frac1{24} \left((x+2)^{2}|x+2| - 2 x^{2}|x| + (x-2)^{2}|x-2|\right) $$

The degree *n* of the smooth absolution function |*x*|_*n*_ introduced above has a smoothing range over the interval [−*n*,*n*], as |*x*|_*n*_=|*x*| when |*x*|>*n*. Smooth absolution functions with an arbitrary smoothing range [−*δ*,*δ*] (*δ*>0) can be easily introduced by using |*x*|_*n*_ in the following way:

8 $$ |x|_{n, \delta}=\frac{\delta}n \left|\frac{nx}{\delta}\right|_{n}.  $$

**Fig. 13:**
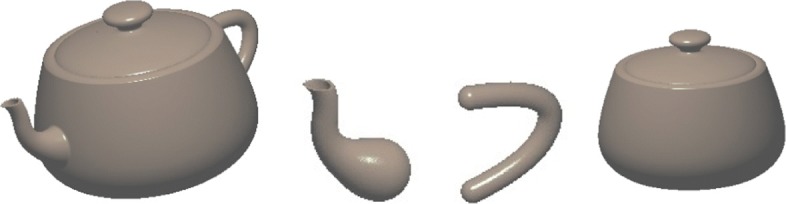
Shape-preserving smooth blending allows to sub-divide a relatively complex object into simpler components, each of which can be designed individually. These individually designed components can then be combined together smoothly by using a smooth shape-preserving implicit function-blending operation

**Fig. 14:**
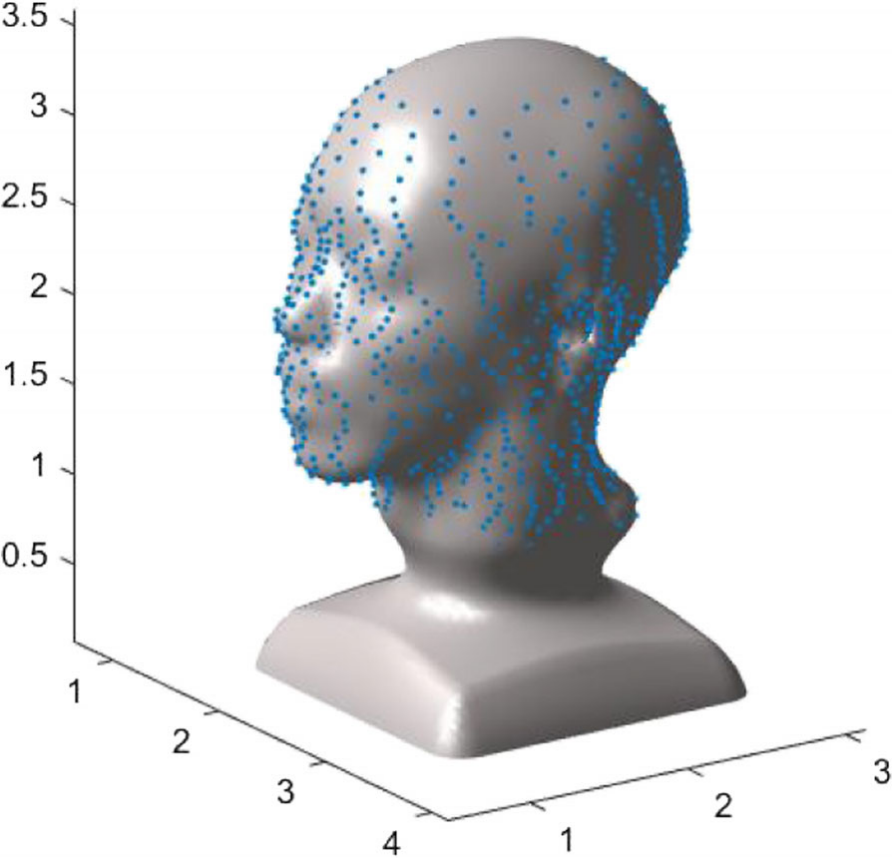
Implicit shape designed by blending an implicit geometry with a real world object reconstructed by using an implicit fitting technique

Figure 13 demonstrates why shape-preserving implicit blending is useful. As it can be seen from the figures, with the availability of shape-preserving blending, any complex geometric shape can be designed implicitly part-by-part and individually as simple geometric primitives, since these independently designed objects can be combined together without changing their original geometries, except for the regions close to the joints of these shapes. The shape-preserving blending becomes even more essential when parts of the objects are reconstructed from a real object, such as the shapes obtained from a certain reverse-engineering technique (Fig. 14).

## Conclusion

With the increasing availability of 3D printers, there is a pressing need to develop 3D printing-oriented geometric modeling techniques. Most conventional CAD techniques are developed based on the need of visualization and traditional subtractive manufacturing, rather than that of the AM, and the geometric objects generated by these techniques are mainly boundary-based and cannot be sent to an AM system for direct printing. The conversion of a surface-represented geometry into a printable representation is in general a complex procedure, and most often a redesign process. In this paper, we have explained and shown why implicit modeling is an ideal geometric object representation for the AM. However, compared with surface-based modeling, much less attention has been paid to implicit geometric modeling. Though there are increasingly more applications of implicit modeling, many open and challenging theoretical and technical issues and problems remain to be solved, which requires a collective effort from mathematicians, computer scientists, AM engineers, and researchers.

It should be noted that while implicit modeling is AM-friendly, it is not a convenient form of subtractive manufacturing, where the boundary of a slice needs to be calculated, which is not a simple task when the internal support structure is relatively complicated [46].

As a conclusion, we put forward some key technical challenges concerning the development of the AM-friendly CAD techniques that urgently need to be overcome.

- *Developing new AM-oriented CAD tools*. The lack of 3D printing ready models is one of the many factors that hugely limits the use of 3D printers. Most existing CAD tools are subtractive manufacturing-oriented, which does not in general fit the use for creation of 3D printing-friendly models. New AM-oriented tools that can represent both shape and material properties are urgently needed [47].
- *Application and person-specific customized implicit modeling*. One typical type of objects that is most suitable to be produced by AM techniques is the application-specific or person-specific customized objects. These bespoke geometric objects are often reconstructed from real objects, from the scanned data, or from a set of pre-specified constraints. This kind of a modeling task is essential, for instance, in creation of geometric models for reconstruction of human organs or in plastic surgeries.
- *Developing implicit shape and material libraries*. One reason why implicit modeling is much less popular than the explicit modeling method is the sparse availability of ready-to-use implicit models. The development of a library containing a rich set of implicit models will definitely boost the use of the implicit modeling technique.
- *Material structure optimized implicit design*. The high cost of printing materials is often considered as one of the top challenges faced by AM. In implicit modeling, more research is required to develop material structure optimization techniques to minimize the use of the printing material.
